# Experiences and mentoring needs of novice nurse educators at a public nursing college in the Eastern Cape

**DOI:** 10.4102/hsag.v25i0.1295

**Published:** 2020-08-24

**Authors:** Khanyisa Annelice Sodidi, Sihaam Jardien-Baboo

**Affiliations:** 1 Lilitha College of Nursing, Eastern Cape Department of Health, Port Elizabeth, South Africa; 2 Department of Nursing Science, Faculty of Health Science, Nelson Mandela University, Port Elizabeth, South Africa

**Keywords:** college, novice educators, novice, expert, proficient, mentoring

## Abstract

**Background:**

When novice nurse educators enter academia, they are expected to demonstrate and implement knowledge in the clinical and classroom environment. However, when one enters academia without proper guidance and support, these expectations create lack of role models. Although mentorship has proved to make the transition easier, there is a lack of mentoring in most nursing schools and/or departments at higher education institutions in South Africa because of scarcity of mentoring programmes for novice nurse educators.

**Aim:**

The aim of this study was to explore and describe the experiences and mentoring needs of novice (newly qualified) nurse educators at a public nursing college in the Eastern Cape, to make recommendations for the mentoring of novice nurse educators.

**Setting:**

Urban and rural public nursing college campuses and sub-campuses in the Eastern Cape.

**Methods:**

Qualitative research approach and exploratory, descriptive, contextual and phenomenological designs were used. Sampling was purposive, data were collected by using semi-structured individual interviews and analysed using Tesch’s method.

**Results:**

Five themes emerged from this study. Findings indicated that novice nurse educators experienced lack of theoretical and clinical mentoring and lack of orientation and resources. Participants also provided recommendations to optimise the experience and performance of novice nurse educators.

**Conclusion:**

Lack of mentoring causes difficult transition by novice nurse educators from the nursing role into the nurse educator role. The implementation of the recommendations on mentoring of novice nurse educators would optimise the experience and performance of the novice nurse educators, thus enhance their smooth transition into academia.

## Introduction

Mentoring is the guidance and support provided to a junior or inexperienced personnel member by a professional, experienced person and can be both formal (assigned) and informal (chosen). The mentor acts as a teacher, friend or guide and is actively involved in the empowerment and professional development of the junior member (eds. Meyer et al. 2013:160).

Mentors can occur in organisational or collective contexts as well as between two individuals. Encounters and networks between mentor and mentee can be of both long and short term through face-to-face, electronic, telephonic and written vehicles (Hunt 2013:198). The major goal of the mentor developmental relationship is the promotion of potential, talent and achievement (Hunt 2013:200). Positive outcomes of mentoring for teachers, student nurses, nurses, the academic institution and the nursing profession include: enhanced career mobility, success and achievement; increased professional, personal and work satisfaction; increased self-confidence and self-esteem; preparation for leadership roles and activities; development of talent and potential; seeking advanced education; increased motivation and productivity; high performance and excellence in practice; increased recruitment and retention rates; empowerment and networking skills; and sustaining a professional legacy (Vance 2010:36).

The nurse educator role is challenging to novice nurse educators and even more so when mentoring is lacking. Novice nurse educators who enter academia for the first time are expected to demonstrate and implement knowledge in the clinical and classroom environment (Kirchoff & Goeres 2011:5). However, when one enters academia without proper guidance and support, these expectations create role strain, stress and frustration. Novice nurse educators need the help of a mentor to navigate the new world of being a nurse educator (Hunt 2013:202). Mentoring has proved to make this transition easier (Seekoe 2014:12).

Seekoe (2014:4) conducted a survey, which highlighted the lack of mentoring programmes for newly appointed nurse educators at schools and/or departments of nursing at higher education institutions in South Africa. In Kirchoff and Goeres (2011:4), Baker concludes that nurse educators, during their first full-time teaching year, should feel that they are being nurtured and provided with the tools they need to be successful in academia.

## Problem statement

In the Eastern Cape, recruiting and retaining newly qualified nurse educators have been a challenge in public nursing colleges. A newly qualified nurse educator is a nurse educator who has a maximum of 12 months’ teaching experience and assumes a new position in an academic setting (Kirchoff & Goeres 2011:9). Experienced nurse educators were either retiring or resigning from the college, resulting in a shortage of nurse educators. To curb the shortage, a strategy was adopted by the college management to recruit young, enthusiastic professional nurses to study nursing education. These professional nurses would be funded by the Department of Health. Upon completion of the course, these professional nurses would then be absorbed in the college to fill the vacant nurse educators’ posts. At the academic staff meetings, experienced nurse educators of the college remarked that these professional nurses lacked the necessary clinical nursing experience to become nurse educators, as they were new in the nursing profession. However, being an expert clinician does not necessarily make someone an expert teacher (Hunt 2013:27). Although some of these nurses may be considered as expert nurse clinicians, occupying a new role as an educator makes them novice nurse educators (Schoening in Brown 2015:14).

The covert effect of these statements was that novice nurse educators were unsupported and unguided by the experienced nurse educators. Consequently, novice nurse educators lacked direction and confidence and were uncertain of their expected roles. The researcher found novice nurse educators mentoring one another to move forward and learning their roles through trial and error (Kirchoff & Goeres 2011:9). The dilemma was compounded by the fact that neither universities nor colleges have sufficient mentoring programmes for novice educators, according to Greyling 2003 (cited in Seekoe 2014:2). This lack of mentoring was identified as the main problem by the researcher and the difficult transition of novice nurse educators prompted the researcher to explore and describe the experiences and mentoring needs of novice nurse educators, with the goal of making recommendations on the mentoring of novice nurse educators.

## Aim

The aim of this study was to explore and describe the experiences and mentoring needs of novice nurse educators at a public nursing college in the Eastern Cape, to make recommendations for the mentoring of novice nurse educators.

## Research design and methods

A qualitative research approach and exploratory, descriptive, contextual and phenomenological designs were used in this study to explore and describe the experiences and mentoring needs of novice nurse educators at a public nursing college in the Eastern Cape.

## Research population

A population is the entire group of persons or objects that is of interest to the researcher and meets the criteria that the researcher is interested in studying (Brink, Van Der Walt & Van Rensburg 2012:131). The population for this study included nurse educators who were appointed at a public nursing college in the Eastern Cape, after completing their nursing education qualification and had between 0 and 4 years’ experience as a nurse educator. As a result of the limited sample of newly qualified nurse educators, the criterion was extended to include those who had 0–4 years’ experience instead of 0-1 year experience, to share their first year experiences when they were newly appointed.

## Sampling

Sampling was purposive and the study was conducted on 16 nurse educators at the urban and rural three main campuses and three sub-campuses of the nursing college in the Eastern Cape as the 16 interviews provided saturated data.

## Recruitment

Nurse educators who met the inclusion criteria were recruited via recruitment letters that were written and dispatched to all six campus and sub-campus heads of the sampled campuses and sub-campuses. The researcher followed these up telephonically with the respective campus and sub-campus heads. The heads passed on the names and contact details of the prospective, voluntary participants to the researcher.

## Data collection

Data collection involves gaining permission, conducting a good quality sampling strategy, developing means for recording information, storing the data and anticipating ethical issues (Creswell 2013:145). The researcher collected data by means of face-to-face, semi-structured individual interviews that were 16 unstructured observations and field notes. The interviews were audio recorded digitally by means of a voice recorder, and the 16 interviews provided saturated data that were then transcribed verbatim. Approximately 45–60 min was allocated per interview session, and all interviews took place in offices identified by the participants at their work places and were all conducive to conduct interviews. Permission was requested by the researcher from the participants to participate in the study and to be interviewed as well as the permission to use a digital audio recorder. The interviews were audio recorded digitally to ensure that all the responses by the participants were correctly captured by the interviewer to transcribe them verbatim and for data analysis. Guidance was received from the supervisor on how to conduct interviews. The researcher made use of silence, nodding, minimal verbal response, probing, paraphrasing and summarising as communicating skills during the interviews. A pilot study was conducted and refinements were made to the interview questions before the subsequent interviews were conducted. The broad questions that were asked from the participants during the interviews were:

1. Tell me about your experiences as a novice nurse educator or when you were a novice nurse educator (both positive and negative).
2. What are or were your mentoring needs as a novice nurse educator?
3. What recommendations would you make to optimise the experience and performance of the novice nurse educators in their first year of teaching at a nursing college?

## Data analysis

Data analysis entails categorising, ordering, manipulating and summarising the data and describing it in meaningful terms (Brink et al. 2012:177). In qualitative research, data collection and data analysis occur simultaneously (Grove, Burns & Grey 2013:280). In this study, the collected data were transcribed in its entirety from the digital recordings by the researcher; the researcher translated any isiXhosa responses by the participants. The researcher made use of an independent coder to interpret and analyse data accurately to ensure trustworthiness. The researcher provided the independent coder with the soft and hard copies of the transcripts containing pseudonyms, together with the proposal that presented the background to the study and amplified the aim and objectives of this study. The researcher and the independent coder simultaneously and independently coded the transcriptions by using Tesch’s method of data analysis and reached consensus after discussing the themes and sub-themes that emerged from the data.

## Pilot interview

A pilot interview was conducted to evaluate the adequacy of the selected research methods and procedures. One nurse educator from a local main campus and one nurse educator from a local sub-campus who met the inclusion criteria were included in the pilot interview. The two broad questions that were asked from the participants during interviews were revised and minor refinements were made. Data collected in the pilot interviews were included in the main study as the interviews were a success.

## Ethical consideration

Any research study must comply with sound ethical practices and standards, and there must be a balance between contributing to science and protecting the rights and dignity of human subjects (Pera & Van Tonder 2012:326). According to Terre Blanche, Durrheim and Painter (2011:61) the essential purpose of ethics is to protect the welfare of research participants. The researcher adhered to the three fundamental ethical principles namely: respect for persons, beneficence and justice. The researcher submitted the research proposal to the Research Ethics Committee of the Nelson Mandela University for ethical approval, which was granted on the 02 December 2016 (Ethical Clearance No. H16-HEA-NUR-054). Permission to conduct the research study was granted by the Superintendent General of the Eastern Cape Department of Health, the Eastern Cape Research Committee and the college principal. The campus and sub-campus heads of all the sampled campuses and sub-campuses received formal letters requesting their permission to gain entry into the sites and to access participants.

Ethical clearance to conduct the study was obtained from Nelson Mandela Metropolitan University on 02 December 2016 (Ethical Clearance No. H16-HEA-NUR-054).

## Trustworthiness

Qualitative research is based on trustworthiness rather than reliability and validity (Brink et al. 2012:126). The methods that support the analysis of qualitative studies, namely, credibility, confirmability, dependability and transferability are Guba and Lincoln’s criteria, as cited in the work of De Vos et al. (2011:419). These criteria were used to assess research data quality and to ensure the trustworthiness of the study. For credibility, confirmability and dependability, the researcher kept her opinions, experiences and values to herself to avoid bias in the collected, interpreted and analysed data. A digital recorder was used during the interviews and field notes written on each contact session and interview to ensure that the researcher was not dependent on her memory for recalling the participants’ responses. All data were transcribed verbatim, and an independent coder analysed the data. Transferability refers to the ability to apply the findings in other contexts or to other participants. The qualitative researcher is not primarily interested in generalising the findings, but rather in defining observations within the specific contexts in which they occur (Brink et al. 2012:173). For transferability, the researcher described the context, study design and methods thoroughly and made use of purposive sampling, thick descriptions and data saturation strategies to enhance transferability. In this study, the recommendations made by the participants for the mentoring of novice nurse educators could be transferred or utilised in other similar contexts to enhance smooth transition of novice nurse educators into the academic environment.

## Results and discussion

The findings indicated that novice nurse educators experience difficulties in transition from the nursing role to the nurse educator role, mainly because of lack of mentoring in the nursing schools in which they were employed. Five themes and nine sub-themes will be elaborated briefly with relevant quotations and substantiating literature discussions.

### Theme 1: Novice nurse educators experience challenges related to their assessment practices in theoretical modules

Novice nurse educators experienced challenges because of the lack of moderation of their assessment practices in theoretical modules.

The novice nurse educators stated that they did neither receive sufficient mentoring or support nor receive constructive feedback in their assessment practices. The participants stated that the test question papers and memoranda that they submitted to their Heads of Departments (supervisors and mentors), prior to the assessment and evaluation of students, were not moderated by the supervisors.

Participants shared that the supervisors would sanction their work without reviewing it and that when they implemented these assessments, errors would sometimes be identified by the students or by the participants themselves. Furthermore, participants sensed that the supervisors denied them feedback, as if they were setting them up for failure.

Guidance and support in the usage of other assessment instruments such as specification tables, mark schedule compilation and the type of template to use were not received from the supervisors, which made it all more difficult for the novice nurse educator. One verbatim quotation illustrates this:

‘[*I*]n fact she was not even checking my work because I wouldn’t say she was checking my work if just going, doesn’t even read the question paper if I’m submitting for test, if I’m submitting a lesson plan she doesn’t even check for it and she doesn’t even make some comments on the question paper and she doesn’t even put her signature on my question paper showing that she has seen it, moderated it, find any corrections or whatever.’ (002.1, MSMCP4:3–4, female, 35 years old)

An individualised inventory of faculty strengths and weaknesses based on the core competencies for nurse educators should be initiated. Efforts can then be undertaken to reduce the stress and anxiety of new faculty by acknowledging their strengths and supporting their weaknesses to fulfil the nurse educator role (Kirchoff & Goeres 2011:90).

#### Sub-theme 1.1: Lack of mentoring leads to unnecessary errors and increased workload

The participants verbalised how the lack of mentoring led to unnecessary errors and an increased workload on their part:

‘[*M*]arking of scripts, it was not a good experience because I was not sure how to mark, I’ve just given scripts to mark, as a result I’ve made lots of errors. Student comes back with scripts because I’ve made lot of errors.’ (0003.1, MSMCP2:1–2, female, 44 years old)

‘[*I*]magine when you write a test, (an exclamation for acknowledging a mistake of saying you instead of they), when they write a test, you have to mark hundred scripts, in the mean, in the mean time you not focusing that you need also to do (i) [*the*] accompaniment, which is, is kind of eh … not ok.’ (01.1.1, MSSCP2:1, female, 42 years old)

Experienced faculty should assist new faculty with test preparation and interpretation. Perhaps reviewing prior exams and examining the results would help alleviate the anxiety associated with this process (Kirchoff & Goeres 2011:92).

Careful consideration should be given to the workload complexity of new faculty. Workload complexity continues to be a problem in the profession and will continue to be so unless adjustments are made in teaching complexity. Because of the documented examples of faculty role strain and stress that have resulted in faculty departing from their positions, both novice and experienced faculty should have reduced workload complexity until they adjust to their new professional environments (Kirchoff & Goeres 2011:90).

### Theme 2: Novice nurse educators experience challenges related to the lack of mentoring in the clinical component of the programme

The participants indicated that the challenges that they experienced included a lack of clinical mentoring and a lack of clinical skills development. Novice nurse educators reported that they were not mentored into the clinical educator’s role and that the clinical procedures were not demonstrated to them. They noticed that the performance of clinical procedures and the clinical evaluation tools used to assess the students was not standardised. In the words of the participants:

‘[*I*] was expected to do the demonstrations on those skills yet I didn’t know, I wasn’t sure whether I was using the correct things to those students, but what I’m saying, you will be corrected sometimes in front of the students, no we do things like this here, you see?’ (01.1.1, MSSCP2:1, female, 42 years old)

A nurse educator requires a different set of skills from those of a registered nurse. Therefore, it is imperative that novice nurse educators are equipped with the proper training to meet the high demands of the nurse educator role (Jacobson & Sherrod 2012:281).

Many nurses who accept an educator position have expert clinical experience; however, as Schoening (in Brown 2015:14) states, these clinicians struggle with becoming acclimated to the academic setting and skills that are parallel to the educator role.

A deficiency in academic knowledge amongst the novice educators can hinder their competencies from being fully executed (Brown 2015:21). Nurse educators, according to Kotzé et al. (2013:201), should have in-depth knowledge and understanding of the part of the curriculum for which they are responsible, so that they are able to promote the success of their students. Tinto (2009:6) states: ‘Faculty are responsible for keeping current with all new information, technology, policies and procedures, and teaching and learning strategies’.

### Theme 3: Novice nurse educators experience a lack of orientation in both the academic and clinical environments

The participants experienced a general lack of orientation to the nursing college regarding their role as nurse educators, the college culture and environment and all the personnel employed at the college. Similarly, the novice nurse educators expected to receive an in-depth orientation to the clinical environment, the clinical area managers and to the staff members. They were disappointed when all they received was a brief introduction to the managers. One participant stated:

‘[*I*] went to clinical area without knowing what is expected of me.’ (01.1.1, MSSCP2:1)

The participants not only reported a lack of orientation and introduction to institutional structure, processes and culture but also a lack of support regarding their orientation needs and expectations. In the words of a participant:

‘[*I*] felt that I needed somebody who can sit with me and go through the policies, specifically policies related to academics.’ (0003.1, MSMCP1:3)

Being unaware of the organisational structure, benefits and promotion, expectations increase the likelihood of an unsuccessful transition (Hunt 2013:180). Kotzé (1998:6) indicates that individuals need to feel safe and secure with regard to environment and relationships with others; individuals want to know that they are wanted and that those around them are interested and concerned.

#### Sub-theme 3.1: Unpleasant emotions experienced during the transition period owing to a lack of orientation

The novice nurse educators experienced unpleasant emotions as a result of lack of orientation such as the frustration of not knowing what was expected of them; they verbalised self-doubt and a lack of confidence in their abilities when they were allocated to teach components about which they had no previous experience. The participants verbalised experiencing culture shock regarding the difference between the way the college functioned, as opposed to how the institutions where they had been employed previously functioned. One participant displayed her frustration:

‘[*I*]t frustrates a lot because there was no communication, yes I realise later on that there was a year plan, but eh eh eh at the beginning I couldn’t eh gel much.’ (0003.1.1, MSSCP2:3)

One of the categories that emerged from the data analysis of a study conducted by Anibas, Brenner and Zorn (2009:214) was ‘feelings’, whereby a variety of feelings were expressed by the participants. These included worry, frustration, uncertainty about own performance, what to expect, confusion, awkwardness, isolation, expendability and fear about patient and student safety. Brown (2015:22) adds that many emotions and feelings are combated within the first few years of teaching.

#### Sub-theme 3.2: Inadequate administrative and management processes

The participants expressed that they experienced inadequacies related to the human resources (HR) department and management. Shortcomings in the HR department included delayed appointment letters for new staff members and the incorrect appointment of staff members. Management inadequacies included: delays in novice nurse educators receiving their job descriptions; infrequent or poor communication about roles and expectations; incorrect clinical allocations; and delays in the distribution of work. Managers and supervisors were largely unavailable to novice nurse educators. The participants described these shortcomings as:

‘[*O*]ur letter of our appointment came very … late, at least after a year when we’ve been fighting.’ (0003.1.1, PSSCP:3)

‘[*A*]fter two months, It’s when I only received the, what is it? The job descriptions.’ (0003.1, PSMCP:10)

According to Kirchoff and Goeres (2011:93), functioning within the education environment can be accomplished with new faculty through a review of job descriptions and expectations of the position. Furthermore, faculty should understand where they fit within the department and larger college or university environment.

#### Sub-theme 3.3: Novice nurse educators experience different forms of exploitation

The novice nurse educators felt that the college management had exploited them when they had to work overtime, but were not remunerated accordingly. Many of the participants lost their service benefits because they were not translated according to the Performance Management and Development System. Participants also reported being exploited by their supervisors by being assigned to duties unrelated to their level of education, such as being a vehicle driver. The statement of a participant expresses this:

‘[*D*]uring that period of two months I was just lying around, sometimes I was asked to help lecturers who would be going to (saying the name of the town), then I, I would eh work as a driver, in that case because almost, almost every week I was asked to drive people to the meetings, to (saying the name of the town) and back.’ (003.1, PSMCP:2)

Exploitation of labour is the act of treating one’s workers unfairly and for one’s benefit. It is a social relationship based on a fundamental asymmetry in a power relationship between workers and their employers (Bala 2008:1).

### Theme 4: Novice nurse educators experience a lack of resources

The lack of resources reported by the participants included: office space, staff members, teaching equipment, student and staff support services, recent and updated editions of books, library, internet, technology and transport facilities.

#### Sub-theme 4.1: Additional barriers to teaching students are created by limited resources

The lack of individualised office space deprived the novice educators of their privacy and they prepared for their tasks in crowded areas with many distractions. There was no private space for the educators to meet students for consultation and counselling. Participants stated that theoretical and clinical components were overwhelming, and the recommended student–lecturer ratio that was not adhered to was justified by citing staff shortage. The limited access to technology, like the Internet, proved challenging to the participants at the college who wanted to prepare lessons and PowerPoint presentations. The limited number of recent and updated textbooks in the campus libraries proved challenging to the novice nurse educators who wanted to prepare lessons and lecture demonstrations. Limited transport resources at the college led to challenges in the transportation of students and nurse educators between the college and the clinical areas. The limited resources were said to be causing additional barriers to the teaching of students. The following verbatim quotes indicate these sentiments:

‘[*T*]he resources are not enough. We don’t have all the resources to assist those students, even myself alone, my own resources, we don’t have computers, I have to consult hospitals so that I can borrow one of the resources that I can use to help the students.’ (002.1.1, MSSCP3:2, female, 50 years old)

The lack of resources is perceived as a barrier to education across cultural, geographical and economic boundaries (Miles 2000:2). This can be seen from the below words of a participant:

‘[*Y*]es there was a library but there were not enough books and the most books in our library were too were too old, so it was making things difficult for us especially when we are doing our lesson plans.’ (002.1, MSMCP4:8, female, 35 years old)

In a study conducted by Matshotyana (2015:53) at the public nursing college under study, the second-year students who were interviewed for the study at the time identified the physical resources lacking on the college campuses as follows: classrooms, computer laboratories, student transport to the clinical practice environments and equipment for performing skills and procedures. Making use of the resource committee is a recommended strategy whereby the committee is responsible for evaluating current resources and making recommendations for improving, updating or adding new resources. These resources relate to the resources faculty need to provide quality education and the resources students need to succeed in the programme (Hunt 2013:188).

### Theme 5: Novice nurse educators provide recommendations to optimise the experience and performance of the novice nurse educators in their first year of teaching at a nursing college

Novice nurse educators had negative experiences in their first year of teaching at the college and expressed the hope that future novice nurse educators would experience the college differently through the recommendations that they propose. The recommendations provided by the participants included the development and implementation of structured, comprehensive orientation and mentoring programmes, improvement in communication and uniformity in the implementation of institutional policies and procedures by management.

#### Sub-theme 5.1: The development and implementation of a structured and comprehensive orientation programme

The participants recommended the development and implementation of a structured and comprehensive orientation programme to optimise the experience and performance of the novice nurse educator in their first year of teaching at a nursing college. As voiced by a participant:

‘[*T*]here should be structured orientation programme that should be followed by the, by the senior nurse educators to assist the new lecturers.’ (01.1.1, MSSCP2:2–3, female, 42 years old)

According to Lilitha College of Nursing (2013:21), a situational analysis indicates that nurse educators face a lack of orientation or induction programmes.

Novice educators need an intense outline of responsibilities, policies and procedures, and teaching expectations, which is considered an orientation (Brown 2015:25).

#### Sub-theme 5.2: The development and implementation of a structured and comprehensive mentoring programme

Participants recommended the development and implementation of a structured and comprehensive mentoring programme to optimise the experiences and performance of the novice nurse educators in their first year of teaching at a nursing college. One of the participants recommended as:

‘[*I*] recommend that we have a mentoring programme in the college and then we … support the new nurse educators.’ (002.1, MSMCP2:5, female, 31 years old)

The National League for the Nursing Board of Governors (in Brown 2015:20) asserts that it is essential for experts to be mentors to share their knowledge, wisdom and experiences to cultivate the novice educator’s contribution to nursing education.

#### Sub-theme 5.3: Improvement in management concerning the communication and uniformity in the implementation of institutional policies and procedures

Novice nurse educators identified gaps in the college management’s communication and implementation of institutional policies and procedures and recommended that there be more uniformity in these areas to optimise the experience and performance of novice nurse educators in their first year of teaching at the college. One such proposal from a participant is:

‘[*M*]anagement needs also to improve on their side, so that they talk one thing and, and they mean what they say, they mustn’t in other because irrespective one is in Transkei and one is in other side, the treatment must be the same, you, things must not be based on discretions, if we talk policies, things must be, must be applied according to policies, not depending on, on discretions because discretions are the ones that create problems.’ (0003.1.1, PSSCP:13, male, 42 years old)

According to Huber (in Meyer et al. 2009:199), policies are guidelines that have been formalised and they direct actions, whereas procedures are descriptions of how to carry out actions. Roussel and Swanburg (2009:151) explain that communication is a human process that involves interpersonal relationships and is important in ensuring organisational effectiveness.

#### Sub-theme 5.4: Further suggestions for optimising college functioning

The novice nurse educators had further suggestions for optimising college functioning, which included the standardisation of clinical procedures and clinical evaluation tools, assurance of quality and minimisation of paper work. The following verbatim shows some of the ideas:

‘[*T*]here is especially in clinical because we must know what to do and when to do and how to do and it must be standardised, when our students are here in our labs, it must be the same in outside hospitals.’ (002.1.1, MSSCP3:4–5, female, 50 years old)

‘[*T*]here should be a person who is assigned to monitor quality or anyone who would be an overseen that it is it.’ (01.1.1, MSSCP2:3, female, 42 years old)

‘[*I*] would recommend that eh the paper work eh be … minimised.’ (0003.1.1, MSSCP2:7, male, 41 years old)

The significance of standardisation of clinical procedures is to avoid unwarranted variation in treatment, and to improve patient care and caregiver accountability as it is the establishment of standards and protocols for caregivers to follow when treating patients (Grant 2016:1).

Quality assurance activities must be coordinated, and quality assurance itself must be an ongoing process; it should not be limited by time and should not be a trial and error or once off exercise (Meyer et al. 2009:312).

Legally, an electronic copy of a document can serve the same purpose as a hard copy, as long as the quality of the scanned copy is good enough to read and is a complete copy of the document. Paperwork can pile up quickly, requiring extra filing cabinets, file room space and employee time to file and organise. The amount of paperwork in an HR office can be easily reduced by using computer technology to scan and store electronic copies of documents (Winston 2012:1).

## Limitations of this study

The following are the limitations of this study:

1. Data were collected from novice nurse educators at a public nursing college. The experiences and mentoring needs of novice nurse educators at private universities were not explored in this study; therefore, the research context may prohibit generalisation.
2. A broader range of views may have been elicited had the study sample included more sub-campuses as the college under study has more sub-campuses than main campuses.

## Recommendations

Based on the research findings and the limitations provided above, the following recommendations for nursing practice, nursing education and nursing research were suggested by the researcher.

### Nursing practice

The following recommendation is proposed for nursing practice:

1. The findings of this research study could be disseminated to the clinical practice environments where the students of the college under study are allocated for clinical exposure. This would inform the clinical nurses and clinical managers of the mentoring needs of novice nurse educators related to clinical practice.

### Nursing education

The following recommendations are proposed for nursing education:

1. The findings of this study could be disseminated by the college managers to the senior nurse educators to inform them of the experiences and mentoring needs of novice nurse educators. The suggestions made by the novice nurse educators regarding their mentoring would facilitate an easier transition to the academic environment.
2. The recommendations for the mentoring programme of novice nurse educators could be disseminated to all public nursing colleges in the country for them to assess, contextualise and implement as there is a recognised lack of mentoring programmes for newly appointed nurse educators at schools and/or departments of nursing at higher education institutions in South Africa.
3. The development and implementation of a structured and comprehensive mentoring programme.

### Nursing research

The following recommendations are proposed for nursing research:

1. A similar study could be conducted at the private universities to obtain a complete picture of the experiences and mentoring needs of novice nurse educators at nursing education institutions in the Eastern Cape.
2. A study could be conducted at the college under study for the evaluation of the effectiveness of the mentoring programme after implementation.

## Conclusion

In South Africa there are many nursing colleges and/or departments of nursing at higher education institutions that are lacking mentoring programmes for newly appointed nurse educators. The latter is one of the causes of difficult transition by nurse educators from the nursing role to the nurse educator role because of the complex demands and expectations of the core roles of nursing education.

The study explored and described the experiences and mentoring needs of novice nurse educators at a public nursing college in the Eastern Cape regarding core roles of nurse educators, to make recommendations for mentoring of novice nurse educators. The implementation of the recommendations on mentoring of novice nurse educators would optimise the experience and performance of the novice nurse educators, thus enhance their smooth transition into academia.
